# New‐Onset Diabetes Mellitus Following Pancreatic Injury: A Systematic Review

**DOI:** 10.1002/wjs.70056

**Published:** 2025-08-19

**Authors:** V. P. C. van Zon, M. C. Tol, P. Krijnen, V. A. L. Huurman, D. A. Ruess, I. B. Schipper

**Affiliations:** ^1^ Department of Trauma Surgery Leiden University Medical Center Leiden the Netherlands; ^2^ Department of Internal Medicine Leiden University Medical Center Leiden the Netherlands; ^3^ Department of Transplant Surgery Leiden University Medical Center Leiden the Netherlands; ^4^ Department of General and Visceral Surgery Center for Surgery Medical Center University of Freiburg Freiburg Germany

**Keywords:** diabetes mellitus, endocrine dysfunction, pancreatic injury, trauma

## Abstract

**Introduction:**

Pancreatic trauma is rare, accounting for only 0.2% of all trauma cases, and reports on long‐term endocrine outcomes are scarce. Patients who undergo pancreatic resection may develop diabetes mellitus.

**Objectives:**

To summarize the available literature reporting the incidence of diabetes mellitus (DM) after pancreatic trauma and to evaluate potential influences on the incidence rates.

**Patients and Methods:**

We searched in PubMed and Embase for studies published between 1990 and July 2025 that enrolled patients with trauma of the pancreas and reported data on new‐onset DM.

**Results:**

Out of 906 identified records, six studies with a total of 297 patients with pancreatic trauma. The overall cumulative incidence of new‐onset DM is 5.7% (95% confidence interval [CI]: 3.4%–9.0%). DM developed in 8.2% (95% CI: 4.4%–13.6%) of patients who underwent (partial) pancreatic resection and in 2.9% (95% CI: 0.8%–7.4%) of patients treated nonoperatively. For low‐grade injuries, the incidence of DM was 5.5% (95% CI: 2.0%–11.5%) compared to 11.1% (95% CI: 4.9%–20.7%) for high‐grade injuries. The time until new‐onset diabetes mellitus was detailed in one study with 15 patients.

**Conclusion:**

Based on the available literature, the estimated overall incidence of DM after pancreatic trauma is 5.7%. High‐grade pancreatic injury seems to increase the risk of developing new‐onset DM, which is possibly influenced by their frequent surgical treatment.

## Graphical abstract

Pancreatic trauma is rare, accounting for only 0.2% of all trauma cases, and reports on long‐term endocrine outcomes are scarce. Patients who undergo pancreatic resection may develop diabetes mellitus. To summarize the available literature reporting the incidence of diabetes mellitus (DM) after pancreatic trauma and to evaluate potential influences on the incidence rates. We searched in PubMed and Embase for studies published between 1990 and July 2025 that enrolled patients with trauma of the pancreas and reported data on new‐onset DM.

## 1 Background

Pancreatic trauma is rare, accounting for only 0.2% of all trauma cases [1]. High‐grade pancreatic injuries (grades III–V) are often treated with partial pancreatic resection [2], resulting in the loss of insulin‐producing beta cells [3]. The loss of beta cells can result in impaired glucose metabolism and diabetes mellitus (DM), which may develop long after the initial injury, partly due to decreasing insulin sensitivity with age [4]. Diabetes is associated with significant long‐term complications, such as retinopathy, nephropathy, and cardiovascular disease [5].

The development of diabetes after pancreatic surgery in patients without pancreatic trauma occurs in 5%–50% of cases and depends on factors such as the type of resection, the volume of the remnant pancreas, and preoperative endocrine function [6, 7]. In these patients, underlying pancreatic disease, such as neoplasm or pancreatitis, was often the indication for the resection. The nature of the underlying disease and its associated treatment is likely to influence the development of new‐onset diabetes mellitus (NODM). In contrast, patients with pancreatic trauma generally have no prior pancreatic disease, which may lead to a different profile of endocrine dysfunction.

Understanding the relationship between pancreas trauma, treatment approaches, and NODM can help to inform patients about their long‐term risks and support personalized treatment strategies. Strategies, such as preserving insulin‐producing beta cells through autologous islet transplantation [8] and routinely assessing endocrine function after trauma, could be explored. However, the incidence and profile of NODM in patients treated for pancreatic trauma remain poorly defined. To address this gap, we conducted a systematic review to assess the incidence of new‐onset diabetes mellitus following pancreatic trauma.

## 2 Methods

This systematic review was conducted in accordance with the Preferred Reporting Items for Systematic reviews and Meta‐Analyses (PRISMA) guidelines [9]. The review was registered in PROSPERO with identifying number CRD420250471575.

### 2.1 Eligibility Criteria

Studies were included if they had reported on patients with pancreatic trauma and DM as an outcome. All included studies had to report on the incidence or evaluation of DM at follow‐up. For studies, including both patients with and without pancreatic injuries, only the results for the former group were considered. Exclusion criteria included case reports, case series with fewer than 10 patients, studies published before 1990, conference abstracts, and reviews. No language restrictions were applied.

### 2.2 Search Strategy

Search strategies targeting pancreatic injury and new‐onset DM were developed in collaboration with a librarian from the Walaeus Library at Leiden University Medical Center (Appendix A). Searches in PubMed and Embase were performed on July 23^th^, 2025. Additionally, reference lists of included studies were reviewed for relevant studies not identified in the initial search.

### 2.3 Selection Process

Search results were uploaded to Covidence (Veritas Health Innovation, Melbourne, Australia) and duplicates were removed. Titles and abstracts, followed by full texts, were screened independently by two reviewers (M.C.T. and V.P.C.v.Z.) to determine eligibility. Disagreements were resolved through discussion.

### 2.4 Data Extraction and Methodological Quality Assessment

Two reviewers (M.C.T. and V.P.C.v.Z.) independently extracted data from the included studies using a standardized data collection form. For one study [10], raw data were provided upon request and included in our analysis. Extracted data included study type, patient population, injury mechanism, DM at baseline, treatment details, follow‐up duration, injury grade, DM diagnostic methods, time to diagnosis, and DM incidence. Severity of pancreatic trauma was classified according to the American Association for the Surgery of Trauma (AAST). Injuries classified according to the Japanese Association for the Surgery of Trauma (JAST) were reclassified according to the AAST for consistency across studies. Pancreas injuries JAST types I, II, and IIIa were classified as AAST grades I–II and JAST type IIIb was classified as AAST grade III or higher [11].

The methodological quality of the included studies was assessed using the “Methodological Index for Non‐Randomized Studies” (MINORS) instrument [12], which evaluates eight items regarding the design of noncomparative studies. Each item is scored as “0” (not reported), “1” (reported but inadequate), or “2” (reported and adequate), with a maximal total score of 16. Two reviewers (M.C.T. and V.P.C.v.Z.) independently scored the methodological quality, and disagreements were resolved through discussion.

### 2.5 Definition of Treatment

Pancreatic resection was defined as total pancreatectomy, distal pancreatectomy (tail resection), pancreatic head resection (Whipple's procedure or pancreaticoduodenectomy), pancreatic body resection, or a combination.

Nonresection management included conservative treatments with or without drain placement, endoscopic retrograde cholangiopancreatography, parenchymal repair, and debridement.

### 2.6 Outcome

The primary outcome was the cumulative incidence of DM following pancreatic trauma. We analyzed the incidence of new‐onset DM across subgroups categorized by treatment type and injury severity.

## 3 Results

**FIGURE 1:**
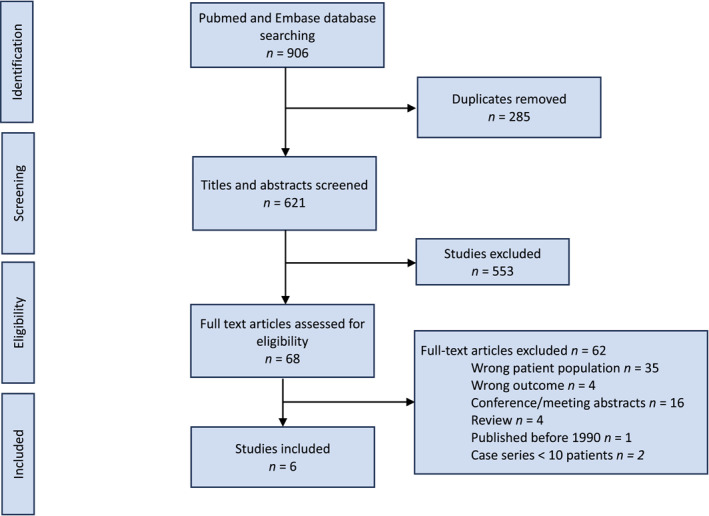
PRISMA flow diagram for the systematic review.

The study selection process is shown in Figure 1. Of 621 unique studies, six articles met the inclusion criteria. Five were retrospective cohort studies [10, 13, 14, 15, 16] and one study included both prospective and retrospective components [17]. Individual studies identified 14 to 165 patients with pancreatic injury, totaling 666 patients. New‐onset diabetes was assessed in 297 of these patients. A total of 222 patients were excluded because the study only included pancreatic surgery [14], 48 were deceased [13, 14, 17], 18 had incomplete data [10, 14], 30 could not be contacted [15, 17], 49 did not give consent [13, 17], and 1 had preexisting diabetes [15]. The outcome was assessed at last clinical follow‐up (*N* = 150) [10], up to 1 year (*N* = 80) [14], up to 4 years (*N* = 18) [15], and after a median of 23 years (*N* = 14) [13]. In one study [17], prospective follow‐up was 6 months (*N* = 17) and retrospective follow‐up was 25.7 months (*N* = 3). The timing of assessment was not reported in one study (*N* = 15) [16]. Penetrating trauma occurred in 31% of the patients and 40% had high‐grade (III–V) pancreatic injuries. Resection was performed on 168 patients (57%), including 126 who underwent distal pancreatectomy. Table 1 summarizes the characteristics of the included studies.

**TABLE 1:** Study characteristics.

Reference	Type of study	Study population	Primary outcome measures	Patients with pancreatic injury	Patients included in analysis (M/F)	Median age (range) at the time of trauma (years)	Mechanism of trauma (*n*)	Treatment (*n*)	Pancreatic injury grade (AAST) (*n*)	Follow‐up
Meijer et al. [10]	Retrospective cohort	Inclusion: — Recent traumatic injury to the pancreas	Patient reported outcomes QoL, endocrine dysfunction, and exocrine dysfunction	165	150	27 (6–93)	Penetrating (15) Blunt (135)	Nonresection: NOM (51) other abdominal surgery (29) Drainage (14) Pancreatic suture (8) Other extra abdominal surgery (1) Resection: DP (32) biliopancreatic diversion (12) Central pancreatic resection (7) PD (2) total pancreatectomy (1) Pancreatic resection NFS (1) N/A (2)	Grade I (36), grade II (59), grade III (46), grade IV (17), grade V (2), and unknown (5)	At last clinical follow‐up
Mansfield et al. [14]	Retrospective cohort	Inclusion: — Patients sustaining pancreatic injury and had pancreatic resection of any type Exclusion: — Nonoperative management — Death before hospital discharge — Preinjury diabetes mellitus — Incomplete or unavailable medical records	Insulin and pancreatic enzyme dependence at the time of discharge If dependent, then follow‐up for endocrine dysfunction	331	80 (70/10)	25 (19–32)	Penetrating (64) Blunt (16)	Nonresection: N/A Resection: DP (73) Proximal pancreatectomy (7)	Not reported	Up to 1 year after pancreatic resection
Rabinovich et al. [16]	Retrospective cohort	Inclusion: — Patients under the age of 18 — Diagnosis of pancreas disease that required invasive procedure Exclusion: — Patients with only endoscopic intervention	Mortality and postoperative complications	15	15	9.5 (1 w 18 y) for the total study group. Not reported for trauma separately	Not reported	Nonresection: Drainage (3) Debridement (3)^a^ Resection: DP (12)	Not reported	Not reported
Morita et al. [13]	Retrospective cohort	Inclusion: — Pancreatic trauma — Informed consent at the time of follow‐up (moment of study) Exclusion: — N/A	Endocrine and exocrine dysfunction	69	14 (10/4)	28.5 (18.5–39)	Blunt (14)	Nonresection: NOM (2) Drainage (2) Repair parenchyma (4) Resection: DP (5) PD (1)	Grade I (0), grade II (4), grade IIIa (3), and grade IIIb (7)^b^	Median 23 y 5 m (range 9 y 1 m–26 y 11 m)
Al‐Ahmadi et al. [15]	Retrospective cohort	Inclusion: — Patients with pancreatic injuries	Functional outcomes not further specified	25	18	35 (24–41)	Penetrating (6) Blunt (12)	Nonresection: No pancreas specific surgery (7) Repair of parenchyma (6) Debridement and packing (1) Resection: DP (4)	Grade I (3), grade II (10), grade III (2), grade IV (2), and grade V (1)	Range 1 m–4 y
Colney et al. [17]	Retrospective cohort (*n* = 3) + prospective cohort (*n* = 17)	Inclusion: — Patients with grades II–V pancreatic injury — Ages 18–65 — Admission in the division trauma surgery and critical care Exclusion: — Preexisting diabetes mellitus — History of pancreatic surgery — History of pancreatitis — Preexisting pancreatic disease — Chronic alcoholics — Death within 30 days of trauma — No informed consent	Endocrine and exocrine function in patients with pancreatic trauma	61	20 (18/2)	30 (18–48)	Penetrating (3) Blunt (17)	Nonresection: NOM (4) Repair of parenchyma (4) Resection: Partial resection of pancreas (12)	Grade II (4), grade III (9), and grade IV (7)	Retrospective (*n* = 3): Mean 25.7 ± 5 months (SD). Prospective (*n* = 17): Six months after pancreatic resection

Abbreviations: DP, distal pancreatectomy; NOM, nonoperative management; PD, pancreaticoduodenectomy; SD, standard deviation.

^a^ 15 trauma patients received a total of 18 procedures.

^b^ Using the JAST 2008 injury score, defined as grades I (subcapsular injury), II (superficial injury), IIIa (simple deep injury), and IIIb (complex deep injury, with main pancreatic duct disruption).

New‐onset DM developed in 17 of 297 patients with traumatic pancreatic injuries (Table 2). The cumulative incidence of DM after pancreatic trauma across studies was 5.7% (95% CI: 3.3%–9.0%), with a range of 0%–35.7% between studies. The diagnosis was made by measured levels of blood glucose and hemoglobin A1c [17], oral glucose tolerance test (OGTT) at 6 months [17], use of insulin at discharge [14], and patient‐reported questionnaires about DM and medication use [10, 13, 15]. One study did not specify how DM was diagnosed [16].

**TABLE 2:** Diabetes incidence, time of diagnosis diabetes, and methods of diagnosis for patients with pancreatic injury.

Reference	Incidence of new‐onset diabetes *n*/*N*	Time to diagnosis diabetes	Methods of diagnosis of diabetes
Meijer et al. [10]	6/150	Not reported	Chart review
Mansfield et al. [14]	2/80	Not reported	Insulin dependence on discharge
Rabinovich et al. [16]	0/15	N/A	Not applicable
Morita et al. [13]	5/14	0 y, 16 y, 19 y, and 24 y (*N* = 2)	Patient‐reported questionnaire about diabetes and medication and HbA1c
Al‐Ahmadi et al. [15]	3/18	Not reported	Patient‐reported questionnaire administered by telephone
Colney et al. [17]	1/20	Not reported	Oral glucose tolerance test (OGTT) at 6 months, HbA1c, and random blood glucose
Total incidence of new‐onset diabetes *n*/*N* (%)	17/297 (5.7%)		

The timing of DM diagnosis was reported in only one study [13]. Of the 14 patients with pancreatic trauma, one patient developed insulin‐dependent DM immediately after sustaining a complex pancreatic injury with duct disruption from a motor vehicle crash and subsequently underwent a pancreaticoduodenectomy. Based on the American Diabetes Association's definition of pancreatogenic diabetes within 5 years after pancreatic injury, only one of 14 patients in the study (7.1%) developed DM within this timeframe. However, additional cases of new‐onset DM were reported at later intervals, specifically at 16, 19, and twice at 24 years postinjury.

Five of the six studies reported the incidence of DM for management with and without (partial) pancreatic resection [10, 13, 15, 16, 17] and one study reported the incidence of DM only in patients who underwent resection [14] (Table 3). The incidence of DM was 2.9% (95% CI: 0.8%–7.4%) in the patients without pancreatic resection and 8.2% (95% CI: 4.4%–13.6%) in patients with (partial) pancreatic resection.

**TABLE 3:** Incidence of diabetes after management with and without (partial) pancreatic resection.

Reference	Management without pancreatic resection			(Partial) pancreatic resection		
	Mechanism of trauma	Type of management	Diabetes incidence	Mechanism of trauma	Type of surgical intervention	Diabetes incidence
Meijer et al. [10]	Penetrating (11)	NOM (51), other abdominal (no pancreatic) surgery (29), drainage (14), pancreatic suture (8), and other extra abdominal surgery (1)	0/103^a^	Penetrating (2)	DP (32), central pancreatic resection (7), biliopancreatic diversion (2), PD (2), pancreatic resection NFS (1), and total pancreatectomy (1)	6/45^a^
	Blunt (92)			Blunt (43)		
Mansfield et al. [14]	N/A	N/A	N/A	Penetrating (64)	DP (73) and proximal resection (7)	2/80^b^
				Blunt (16)		
Rabinovich et al. [16]	Not reported	Debridement and external drainage (3)	0/3	Not reported	DP (12)	0/12
Morita et al. [13]	Blunt (8)	NOM (2), drainage (2), and repair of parenchyma (4)	2/8^c^	Blunt (6)	DP (5) and PD (1)	3/6^c^
Al‐Ahmadi et al. [15]	Penetrating (5)	No pancreas specific surgery (7), repair of parenchyma (6), and debridement and packing (1)	2/14^d^	Penetrating (1)	DP (4)	1/4^d^
	Blunt (9)			Blunt (3)		
Colney et al. [17]	Not reported	NOM (4) and nonpancreas specific surgery (4)	0/8	Not reported	DP and splenectomy (9), DP (2), and near total pancreatectomy (1)	1/12
Total incidence of new‐onset diabetes *n*/*N* (%)			4/136 (2.9%)	Total incidence of new‐onset diabetes *n*/*N* (%)		13/159 (8.2%)

Abbreviations: DP, distal pancreatectomy; NOM, nonoperative management; PD, pancreaticoduodenectomy.

^a^ Outcomes from the chart review, excluded (*N* = 2) having no data on management of injury (*N* = 148). Two new‐onset diabetes occurred after nonresection: two blunt injuries managed with a drain. Two new‐onset occurred after resection: one after DP one after central pancreatectomy both for blunt injuries.

^b^ Two new‐onset diabetes after distal resection and two after proximal resection.

^c^ One new‐onset diabetes after drainage, one after repair of parenchyma (nonresection), one after DP, and one after PD (resection).

^d^ One new‐onset diabetes after nonpancreas specific surgery, one after debridement and packing, and one after distal pancreatectomy.

Three studies (*n* = 182) [10, 13, 15] reported on injury severity of the pancreas in relation to development of DM [10, 13, 15]. For patients with low‐grade injuries (*N* = 110), the incidence of diabetes mellitus (DM) was 5.5% (95% CI: 2.0%–11.5%) and 11.1% (95% CI: 4.9%–20.7%) for patients with high‐grade injuries (Table 4).

**TABLE 4:** Incidence of diabetes mellitus for low‐grade (AAST grades I–II) and high‐grade pancreatic trauma (AAST grades III–V).

Reference	AAST grades I–II			AAST grades III–V		
	Mechanism of trauma	Management	Diabetes outcome	Mechanism of trauma	Management	Diabetes outcome
Meijer et al. [10]	Penetrating (10) and blunt (80)	NOM (40), other abdominal surgery (24), drainage (10), DP (6), pancreatic suture (6), N/A (2), biliopancreatic diversion (1), and pancreatic resection NFS (1)	2/90^b^	Penetrating (5) and blunt (55)	DP (26), NOM (11), central pancreatic resection (7), other abdominal surgery (5), drainage (4), pancreatic suture (2), PD (2), biliopancreatic diversion (1), other extra abdominal surgery (1), and total pancreatectomy (1)	4/60^b^
Morita et al. [13]	Blunt (7)	NOM (1), drainage (1), repair of parenchyma (3), and distal pancreatectomy (2)	3/7^a^	Blunt (7)	NOM (1), drainage (1), repair of parenchyma (1), DP (3), and PD (1)	2/7^a^
Al‐Ahmadi et al. [15]	Penetrating (4) and blunt (9)	No pancreas specific surgery (7) Repair of parenchyma (6)	1/13^c^	Penetrating (2) and blunt (3)	DP (4) and debridement and packing (1)	2/5^c^
Total incidence of new‐onset diabetes *n*/*N* (%)			6/110 (5.5%)	Total incidence of new‐onset diabetes *n*/*N* (%)		8/72 (11.1%)

Abbreviations: DP, distal pancreatectomy; NOM, nonoperative management; PD, pancreaticoduodenectomy.

^a^ Using the JAST 2008 injury score, defined as grades I (subcapsular injury), II (superficial injury), IIIa (simple deep injury), and IIIb (complex deep injury; with main pancreatic duct disruption). JAST IIIb is equivalent to AAST grade III or higher. One new‐onset diabetes occurred after (drainage), one after (repair of parenchyma), and one after (distal pancreatectomy). Another two new‐onset diabetes occurred after grade JAST IIIb (one distal pancreatectomy and one pancreaticoduodenectomy).

^b^ Outcomes from the chart review (*n* = 150). Two new‐onset DM in AAST I–II. One occurred after pancreatic resection NFS and one after DP. Four had new‐onset DM in AAST III–V and three after DP and one after TP.

^c^ One new‐onset diabetes occurred after NOM (grade II). 5/18 patients (grade I–II) were not evaluated. Two new‐onset diabetes occurred in grade III (DP) and grade V (debridement). 1/7 (grades III–V) was not evaluated for new‐onset diabetes. 1/7 had preexisting diabetes.

### 3.1 Methodological Quality Assessment

The methodological quality of one study [16] was rated as poor according to the MINORS tool [12], with a score of 4 out of 16 (Table 5). The other studies [10, 13, 14, 15, 17] were of moderate quality with MINORS scores ranging from 9 to 11 out of 16 (Table 5).

**TABLE 5:** Methodological quality of included studies assessed according to the methodological index for nonrandomized studies (MINORS) instrument.

	Meijer et al. [10]	Mansfield et al. [14]	Rabinovich et al. [16]	Morita et al. [13]	Al‐Ahmadi et al. [15]	Colney et al. [17]
1. A clearly stated aim	2	2	1	2	2	2
2. Inclusion of consecutive patients	2	2	2	1	2	2
3. Prospective collection of data^a^	2	1	1	1	2	2
4. Endpoints appropriate to the aim of the study	1	1	0	2	2	2
5. Unbiased assessment of the study endpoint	0	0	0	0	0	0
6. Follow‐up period appropriate to the aim of the study	2	1	0	2	0	2
7. Loss to follow‐up less than 5%	2	2	0	1	1	1
8. Prospective calculation of the study size	0	0	0	0	0	0
Total	11	9	4	9	9	11

*Note:* The items are scored 0 (not reported), 1 (reported but inadequate), or 2 (reported and adequate). Maximum score is 16 for noncomparative studies. A score of < 8 was considered to poor quality, 9–14 moderate quality, and 15–16 good quality for noncomparative studies [9].

^a^ Data were collected according to a protocol established before the beginning of the study.

## 4 Discussion

Reports on long‐term endocrine function after pancreatic injury remain limited. In this systematic review, we sought to assess the incidence of DM following pancreatic resection and other management strategies for pancreatic injuries. Six studies on this topic were identified. These studies were, at best, only moderately suited to answer our research question, and it is likely that bias was introduced. Patient selection, inclusion criteria, diagnostic methods, and follow‐up times varied across studies, limiting the ability to draw definitive conclusions about new‐onset diabetes in a specific patient population. The included studies had limited patient selection to address the research question and represent all trauma patients with pancreatic injury, as some only included grade II–V injuries [17] or assessed those requiring insulin at discharge [14], leading to the exclusion of many patients. Additionally, a significant limitation was that only about half of the patients with pancreatic injuries were investigated for diabetes outcomes [13, 14, 17].

Based on the data obtained in this review, we found a cumulative incidence of DM of 5.7% following pancreatic injury. Low‐grade pancreatic injuries were associated with a cumulative DM incidence of 5.5%, whereas high‐grade injuries were associated with an incidence of 11.1%. Low‐grade pancreatic injuries are generally managed without resection, whereas high‐grade injuries are more often treated with partial resection of the pancreas [18, 19, 20]. The cumulative incidence of DM after management without (partial) pancreatic resection was estimated to be 2.9% and 8.2% after partial pancreatic resection. The incidence of DM after partial resection of the pancreas appears higher than in patients who underwent nonresection management. The risk of developing new‐onset diabetes increases as more endocrine mass is resected [21, 22, 23]. De Bruijn et al. [24] reported a 14% cumulative incidence of new‐onset DM after distal pancreatectomy (DP) for benign or potentially malignant lesions and 39% for chronic pancreatitis. Other studies found a 36% DM incidence 2 years after DP [25] and 74.1% after 36 months [26]. This is higher than the incidence reported in our review. These differences may be in part explained by standardized testing and follow‐up of all patients at set time points.

The methods used to diagnose new‐onset diabetes varied widely, likely leading to an underestimation of its true incidence. One study reliably used blood glucose, HbA1c levels, and an OGTT at 6 months [17] whereas others relied on DM medication use at discharge [14] or self‐reported questionnaires [10, 13, 15]. One study [16] did not specify its criteria. This variability likely contributed to differences in reported outcomes, with objective tests are generally preferred for diagnostic accuracy and questionnaires serving as alternatives when standardized testing is not feasible.

Although these findings provide some insight into new‐onset diabetes after pancreatic injury, they should be interpreted with caution. The available studies lacked predefined time points for follow‐up, had insufficient follow‐up durations, experienced high loss to follow‐up, and often used inadequate diagnostic methods.

The study by Morita [13] was the only study that reported the time to diagnosis of DM. Although these patients were relatively young at the time of their DM diagnosis, the influence of the initial pancreatic trauma and its treatment on the development of DM years later remains unclear.

Although some of the included studies [13, 14, 15] reported on exocrine pancreatic insufficiency (EPI), this outcome was not the focus of our review, despite its potential impact on glycemic control through altered incretin secretion. Evidence from chronic pancreatitis and cystic fibrosis populations suggests that pancreatic enzyme replacement therapy (PERT) can enhance postprandial incretin and insulin responses [5], highlighting the need for future studies to evaluate both exocrine and endocrine outcomes following pancreatic trauma.

Determining the true incidence of DM following pancreatic injury remains challenging. To validate and generalize current findings, future research should prioritize rigorous patient selection, detailed characterization of patient cohorts, and robust methodologies. Studies should also include larger sample sizes and extended follow‐up periods of at least 5 years to align with the timeframe for pancreatogenic DM, especially given that trauma often affects younger patients with preserved endocrine reserve. We recommend that further studies use one or more of the following criteria to diagnose diabetes mellitus postinjury: fasting plasma glucose, two‐hour plasma glucose during a 75‐g oral glucose tolerance test, hemoglobin A1c, or random glucose accompanied by classic hyperglycemic symptoms/crises criteria [27]. To fully inform patients after their injury, future studies should examine the time to DM onset in trauma patients and compare outcomes across surgical approaches, considering pancreatic endocrine cell distribution, particularly the higher concentration of insulin‐producing beta cells in the tail of the pancreas [28] to clarify links between resection type and DM risk. Additionally, future research should include a control group of patients without pancreatic injury to more accurately assess the impact of pancreatic injury on the development of diabetes.

This review is limited by the scarcity of research in this field. Pancreatic injuries are rare, and because trauma surgeons primarily manage acute care, long‐term complications, such as diabetes mellitus, are often diagnosed and addressed later by other healthcare providers. Although the incidence of diabetes mellitus may be underestimated, this review represents an initial step toward greater recognition of the long‐term consequences of diabetes mellitus following pancreatic injury.

## 5 Conclusion

There is limited research on diabetes following pancreatic injury, with poor methodological quality in studies investigating new‐onset diabetes postpancreatic injury. Based on the available literature, we reported a cumulative incidence of diabetes mellitus after pancreatic trauma of 5.7%. However, the exact incidence of DM following pancreatic injury remains unclear. High‐grade pancreatic injury seems to increase the risk of developing new‐onset DM, which may be influenced by the higher likelihood of surgical treatment in these cases.

## Author Contributions

**V.P.C. van Zon:** conceptualization, methodology, investigation, formal analysis, writing – original draft, writing – review and editing, visualization, project administration. **M.C. Tol:** conceptualization, methodology, investigation, formal analysis, writing – original draft, writing – review and editing, visualization, project administration. **P. Krijnen:** conceptualization, methodology, writing – original draft, writing – review and editing, supervision. **V.A.L. Huurman:** writing – review and editing. **D.A. Ruess:** writing – review and editing. **I.B. Schipper:** conceptualization, methodology, writing – original draft, writing – review and editing, project administration, supervision.

## Conflicts of Interest

The authors declare no conflicts of interest.

## Supporting information


Supporting Information S1

## Appendix A Search Strategy

### PubMed

(“Pancreas trauma” [tiab:∼3] OR “pancreatic trauma” [tiab:∼3] OR “pancreas injury” [tiab:∼3] OR “pancreatic injury” [tiab:∼3] OR “pancreas injuries” [tiab:∼3] OR “pancreatic injuries” [tiab:∼3] OR “pancreatic laceration” [tiab:∼3] OR “lacerated pancreas” [tiab:∼3] OR “Pancreaticoduodenectomy” [Mesh] OR “Pancreatectomy” [Mesh] OR “Pancreaticoduodenectom*” [tiab] OR “Pancreatectom*” [tiab] OR “pancreas surgery” [tiab:∼3] OR

“pancreatic surgery” [tiab:∼3] OR “pancreas surgeries” [tiab:∼3] OR

“pancreatic surgeries” [tiab:∼3] OR “Blunt abdominal trauma” [tiab] OR “Wounds, Nonpenetrating” [Mesh] OR “Nonpenetrating abdominal” [tiab] OR “pancreatic Resection” [tiab] OR “partial pancreas resection” [tiab]) AND (“Diabetes Mellitus” [Mesh] OR “Diabetes Mellitus” [tiab] OR “Glucose Intolerance” [Mesh] OR “Glucose Intolerance*” [tiab] OR “hyperglycemia” [tiab] OR “hyperglycemic” [tiab] OR “glucose metabolism disorder*” [tiab] OR “pancreatic failure” [tiab] OR “pancreatogenic diabetes” [tiab] OR “Type 3C” [tiab]) AND (“adverse effect*” [tiab] OR “Long Term Adverse Effects” [Majr] OR “prevalence” [ti] OR “incidence” [ti] OR “long‐term outcome*” [tiab] OR “Epidemiology” [Majr] OR “Epidemiology” [ti] OR “Causality” [Majr] OR “Causalit*” [tiab] OR “etiolog*” [tiab] OR “Morbidity” [Majr] OR “Morbidity” [ti])

### Embase

1. ((Pancreas adj3 trauma) or (pancreatic adj3 trauma) or (pancreas adj3 injury) or (pancreatic adj3 injury) or (pancreas adj3 injuries) or (pancreatic adj3 injuries) or (pancreatic adj3 laceration) or (lacerated adj3 pancreas)).mp. or “exp Pancreaticoduodenectomy”/or “exp Pancreatectomy”/or “Pancreaticoduodenectom*”.ti,ab. or “Pancreatectom*”.ti,ab. or “pancreas surgeryADJ3”.mp. or (pancreatic adj3 surgery).mp. or (pancreas adj3 surgeries).mp. or (pancreatic adj3 surgeries).mp. or “Blunt abdominal trauma”.ti,ab. or “exp Blunt Trauma”/or “Nonpenetrating abdominal”.ti,ab. [mp = title, abstract, heading word, drug trade name, original title, device manufacturer, drug manufacturer, device trade name, keyword heading word, floating subheading word, and candidate term word] 47,317
2. (((Pancreas adj3 trauma) or (pancreatic adj3 trauma) or (pancreas adj3 injury) or (pancreatic adj3 injury) or (pancreas adj3 injuries) or (pancreatic adj3 injuries) or (pancreatic adj3 laceration) or (lacerated adj3 pancreas)).mp. or “exp Pancreaticoduodenectomy”/or “exp Pancreatectomy”/or “Pancreaticoduodenectom*”.ti,ab. or “Pancreatectom*”.ti,ab. or “pancreas surgeryADJ3”.mp. or (pancreatic adj3 surgery).mp. or (pancreas adj3 surgeries).mp. or (pancreatic adj3 surgeries).mp. or “Blunt abdominal trauma”.ti,ab. or “exp Blunt Trauma”/or “Nonpenetrating abdominal”.ti,ab.) and (“exp Diabetes Mellitus”/or “Diabetes Mellitus”.ti,ab. or “exp Glucose Intolerance”/or “Glucose Intolerance*”.ti,ab. or “hyperglycemia”.ti,ab. or “hyperglycemic”.ti,ab. or “glucose metabolism disorder*”.ti,ab. or “pancreatic failure”.ti,ab. or “pancreatogenic diabetes”.ti,ab. or “Type 3C”.ti,ab.) and (“adverse effect*”.ti,ab. or “Long Term Adverse Effects”.mp. or “prevalence”.ti,ab. or “incidence”.ti,ab. or “long‐term outcome*”.ti,ab. or “exp Epidemiology”/or “Epidemiology”.ti,ab. or “exp Causality”/or “Causalit*”.ti,ab. or “etiolog*”.ti,ab. or “exp Morbidity”/or “Morbidity”.ti,ab.) [mp = title, abstract, heading word, drug trade name, original title, device manufacturer, drug manufacturer, device trade name, keyword heading word, floating subheading word, and candidate term word] 598
3. “exp Diabetes Mellitus”/or “Diabetes Mellitus”.ti,ab. or “exp Glucose Intolerance”/or “Glucose Intolerance*”.ti,ab. or “hyperglycemia”.ti,ab. or “hyperglycemic”.ti,ab. or “glucose metabolism disorder*”.ti,ab. or “pancreatic failure”.ti,ab. or “pancreatogenic diabetes”.ti,ab. or “Type 3C”.ti,ab. 429,528
4. “adverse effect*”.ti,ab. or “Long Term Adverse Effects”.mp. or “prevalence”.ti,ab. or “incidence”.ti,ab. or “long‐term outcome*”.ti,ab. or “exp Epidemiology”/or “Epidemiology”.ti,ab. or “exp Causality”/or “Causalit*”.ti,ab. or “etiolog*”.ti,ab. or “exp Morbidity”/or “Morbidity”.ti,ab. 3,737,843
5. 1 and 3 and 4 598
6. ((Pancreas adj3 trauma) or (pancreatic adj3 trauma) or (pancreas adj3 injury) or (pancreatic adj3 injury) or (pancreas adj3 injuries) or (pancreatic adj3 injuries) or (pancreatic adj3 laceration) or (lacerated adj3 pancreas)).mp. or “exp Pancreaticoduodenectomy”/or “exp Pancreatectomy”/or “Pancreaticoduodenectom*”.ti,ab. or “Pancreatectom*”.ti,ab. or (pancreas adj3 surgery).mp. or (pancreatic adj3 surgery).mp. or (pancreas adj3 surgeries).mp. or (pancreatic adj3 surgeries).mp. or “Blunt abdominal trauma”.ti,ab. or “exp Blunt Trauma”/or “Nonpenetrating abdominal”.ti,ab. [mp = title, abstract, heading word, drug trade name, original title, device manufacturer, drug manufacturer, device trade name, keyword heading word, floating subheading word, and candidate term word] 51,165
7. 3 and 4 and 6 635
8. ((Pancreas adj3 trauma) or (pancreatic adj3 trauma) or (pancreas adj3 injury) or (pancreatic adj3 injury) or (pancreas adj3 injuries) or (pancreatic adj3 injuries) or (pancreatic adj3 laceration) or (lacerated adj3 pancreas)).ti,ab. or “exp Pancreaticoduodenectomy”/or “exp Pancreatectomy”/or “Pancreaticoduodenectom*”.ti,ab. or “Pancreatectom*”.ti,ab. or (pancreas adj3 surgery).ti,ab. or (pancreatic adj3 surgery).ti,ab. or (pancreas adj3 surgeries).ti,ab. or (pancreatic adj3 surgeries).ti,ab. or “Blunt abdominal trauma”.ti,ab. or “exp Blunt Trauma”/or “Nonpenetrating abdominal”.ti,ab. 45,570
9. “exp Diabetes Mellitus”/or “Diabetes Mellitus”.ti,ab. or “exp Glucose Intolerance”/or “Glucose Intolerance*”.ti,ab. or “hyperglycemia”.ti,ab. or “hyperglycemic”.ti,ab. or “glucose metabolism disorder*”.ti,ab. or “pancreatic failure”.ti,ab. or “pancreatogenic diabetes”.ti,ab. or “Type 3C”.ti,ab. 429,528
10. “adverse effect*”.ti,ab. or “Long Term Adverse Effects”.mp. or “prevalence”.ti. or “incidence”.ti. or “long‐term outcome*”.ti,ab. or “exp* Epidemiology”/or “Epidemiology”.ti. or “exp* Causality”/or “Causalit*”.ti,ab. or “etiolog*”.ti,ab. or “exp* Morbidity”/or “Morbidity”.ti. 1,337,321
11. “exp* Diabetes Mellitus”/or “Diabetes Mellitus”.ti,ab. or “exp* Glucose Intolerance”/or “Glucose Intolerance*”.ti,ab. or hyperglycemia.ti,ab. or hyperglycemic.ti,ab. or “glucose metabolism disorder*”.ti,ab. or “pancreatic failure”.ti,ab. or “pancreatogenic diabetes”.ti,ab. or “Type 3C”.ti,ab. 429,528
12. “adverse effect*”.ti,ab. or “Long Term Adverse Effects”.mp. or prevalence.ti. or incidence.ti. or “long‐term outcome*”.ti,ab. or exp*Epidemiology/or Epidemiology.ti. or exp*Causality/or Causalit*.ti,ab. or etiolog*.ti,ab. or exp*Morbidity/or Morbidity.ti. 1,337,321
13. 8 and 11 and 12 199
